# The clinical value of miRNA-21 in cervical cancer: A comprehensive investigation based on microarray datasets

**DOI:** 10.1371/journal.pone.0267108

**Published:** 2022-04-29

**Authors:** Zhi-Min Deng, Gan-Hong Chen, Fang-Fang Dai, Shi-Yi Liu, Dong-Yong Yang, An-Yu Bao, Yan-Xiang Cheng

**Affiliations:** 1 Department of Obstetrics and Gynecology, Renmin Hospital of Wuhan University, Wuhan, Hubei, China; 2 Department of Clinical laboratory, Renmin Hospital of Wuhan University, Wuhan, Hubei, China; 3 Department of Pathology, The People’s Hospital of Honghu, Honghu, Hubei, China; Indian Institute of Technology Delhi, INDIA

## Abstract

Previous work has demonstrated that the expression of microRNA-21 (miR-21) is implicated in cervical cancer (CC). However, little is known regarding its associations with clinical parameters. We first conducted a meta-analysis using data from Gene Expression Omnibus (GEO) microarrays and The Cancer Genome Atlas (TCGA). Then, enrichment analysis and hub gene screening were performed by bioinformatic methods. Finally, the role of the screened target genes in CC was explored. According to the meta-analysis, the expression of miR-21 in cancer tissues was higher than in adjacent nontumor tissues (*P* < 0.05). In addition, 46 genes were predicted as potential targets of miR-21. After enrichment analyses, it was detected that these genes were enriched in various cancer pathways, including the phosphatidylinositol signaling system and mammalian target of rapamycin (mTOR) signaling pathway. In this study, bioinformatic tools and meta-analysis validated that miR-21 may function as a highly sensitive and specific marker for the diagnosis of CC, which may provide a novel approach to the diagnosis and treatment of CC.

## 1. Introduction

Cervical cancer (CC) is one of the most common gynecological carcinomas worldwide, resulting in an unacceptably high mortality rate. Despite continuous advances in the early screening and treatment of CC, its incidence has shown a younger trend in recent years [1]. In addition, the prognoses of patients with CC are still poor. Hence, revealing the molecular characteristics underlying CC and exploring potential therapeutic targets is imperative.

MicroRNAs (miRNAs), a group of small noncoding RNAs, are thought to regulate the expression of a large number of protein-coding genes and are implicated in a variety of biological processes [2]. MicroRNA-21 (miR-21), an oncogenic microRNA involved in angiogenesis, tumor invasion, and tumor metastasis, was proved to be altered in a variety of cancers, such as gastric cancer, non-small cell lung cancer, bladder cancer, prostate cancer, pancreatic cancer, glioblastoma, and breast cancer [3–9], etc. In CC, a substantial body of evidences had verified that miR-21 was differentially expressed and regarded as a potential therapeutic target [10, 11]. However, little is known regarding its association with clinical parameters.

Here, we first calculated the expression of miR-21 in CC based on data from Gene Expression Omnibus (GEO) microarrays and The Cancer Genome Atlas (TCGA). And then, diagnostic meta-analysis was used to clarify the diagnostic value of miR-21. What’s more, bioinformatic analyses, containing Kyoto Encyclopedia of Genes and Genomes (KEGG), Gene Ontology (GO), and protein-protein interaction (PPI) network analysis, were operated to mining the underlying mechanisms of miR-21. In a word, meta-analysis and further bioinformatic techniques were aimed to explore the role of miR-21 in CC.

## 2. Materials and methods

### 2.1 Data collection and the expression of miR-21

A microarray search in CC was conducted in the GEO database (https://www.ncbi.nlm.nih.gov/geo/). Those datasets without expression of miR-21 were excluded. Next, the expression matrix and clinical parameter data for miR-21 were extracted from TCGA (https://cancergenome.nih.gov/). Correlations between the clinical parameters and miR-21 were calculated by IBM SPSS 22.0. The number, mean (M), and standard deviation (SD) of control and experimental group were obtained based on the expression profile information. The expression of miR-21 in each study were visualized by plotting scatter diagrams in GraphPad Prism 8.

### 2.2 Comprehensive meta-analysis

Meta-analysis was carried out under the R environment using *Meta* package. The chi-squared test of *Q* and the *I*^*2*^ statistic were adopted to assess the potential heterogeneity among the studies. Within groups, comparisons were carried out by t-tests. The ratio of the count data was expressed as percentage (%) and compared using *χ2* tests. In addition, the model used in the statistical heterogeneity was decided according to the *I*^*2*^ and *P* value in forest plots. Generally, the random-effect model was adopted when *I*^*2*^ > 50% or *P* < 0.05; otherwise, the fixed-effects model was adopted [12]. To explore the potential sources of heterogeneity, sensitivity analysis was also conducted.

To assess the diagnostic value of miR-21 for CC, the pooled specificity, sensitivity, positive likelihood ratio (PLR), negative likelihood ratio (NLR), and diagnostic odds ratio (DOR) were computed by meta-Disc software. Furthermore, summary receiver operating characteristic (sROC) curves were also drawn in meta-Disc, whereas the ROC curves of each study were plotted in GraphPad Prism. *P* < 0.05 was considered to be meaningful.

### 2.3 Latent targets of miR-21 in CC and the enrichment analysis

On the one hand, the mRNA targets of miR-21 were predicted by the online database miRWalk2.0 (http://zmf.umm.uni-heidelberg.de/apps/zmf/mirwalk2/) [13]. Only those genes projected by more than 6 of the servers were included. On the other hand, the highly expressed genes in CC were downloaded from GEPIA2 (http://gepia2.cancer-pku.cn/) [14]. The intersection of the genes from the two sources were recognized as targeted genes of miR-21. This procedure was implemented in Vennny 2.1.0 (https://bioinfogp.cnb.csic.es/tools/venny/index.html).

Subsequently, the enriched pathways of those genes were analyzed using R software [15, 16]. The circular plot of the KEGG pathways was plotted by an online platform for data analysis and visualization (http://www.bioinformatics.com.cn). And then, String (http://string-db.org/cgi/input.pl) software was used to construct and visualize the protein-protein interaction (PPI) network between those genes [17]. Based on the PPI network, the hub nodes were investigated by the maximal clique centrality (MCC) algorithm in CytoHubba, a plug-in in Cytoscape [18]. According the value of MCC, the top 4 were defined as hub genes.

### 2.4 The expression of hub genes and further validation

Given that four genes were identified, their expression boxplots and disease-free survival (DFS) curves were downloaded from GEPIA2 initially. Subsequently, the correlations between miR-21 and the identified hub genes were verified via LinkedOmics (http://www.linkedomics.org/) [19]. Both these two steps were aimed to shrink the scope of genes and further obtain final targets.

We next validated the miR-21 binding site on targets via miRactDB database on the one hand [20], and GeneCards (http://www.genecards.org/#) were applied to connect the final targets with the interested KEGG enriched pathways on the other hand [21]. In detail, the genes involved in the interested pathways were collected, and then the STRING were performed to constructed the PPI network between those final targets and pathway genes.

## 3. Results

### 3.1 The expression of miR-21 in GEO microarrays

**Fig 1:**
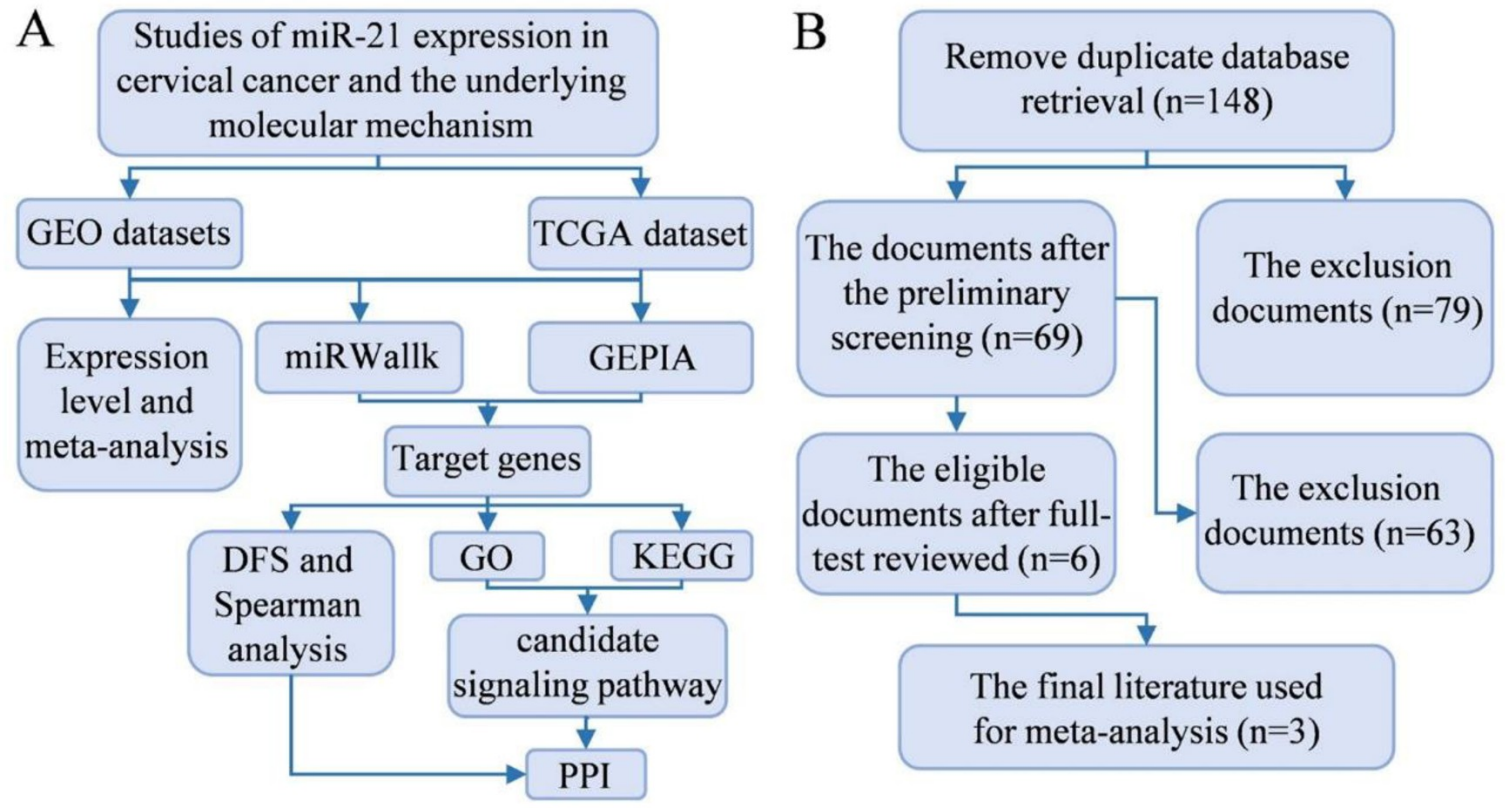
**(A)** The flow diagram of this study. **(B)** Flow chart of the literature screening process and results. Note: miR-21, microRNA-21; GEO, Gene Expression Omnibus; TCGA, The Cancer Genome Atlas; GEPIA, Gene Expression Profiling Interactive Analysis; DFS, disease-free survival; GO, Gene Ontology; KEGG, Kyoto Encyclopedia of Genes and Genomes; PPI, protein-protein interaction network.

**Fig 2:**
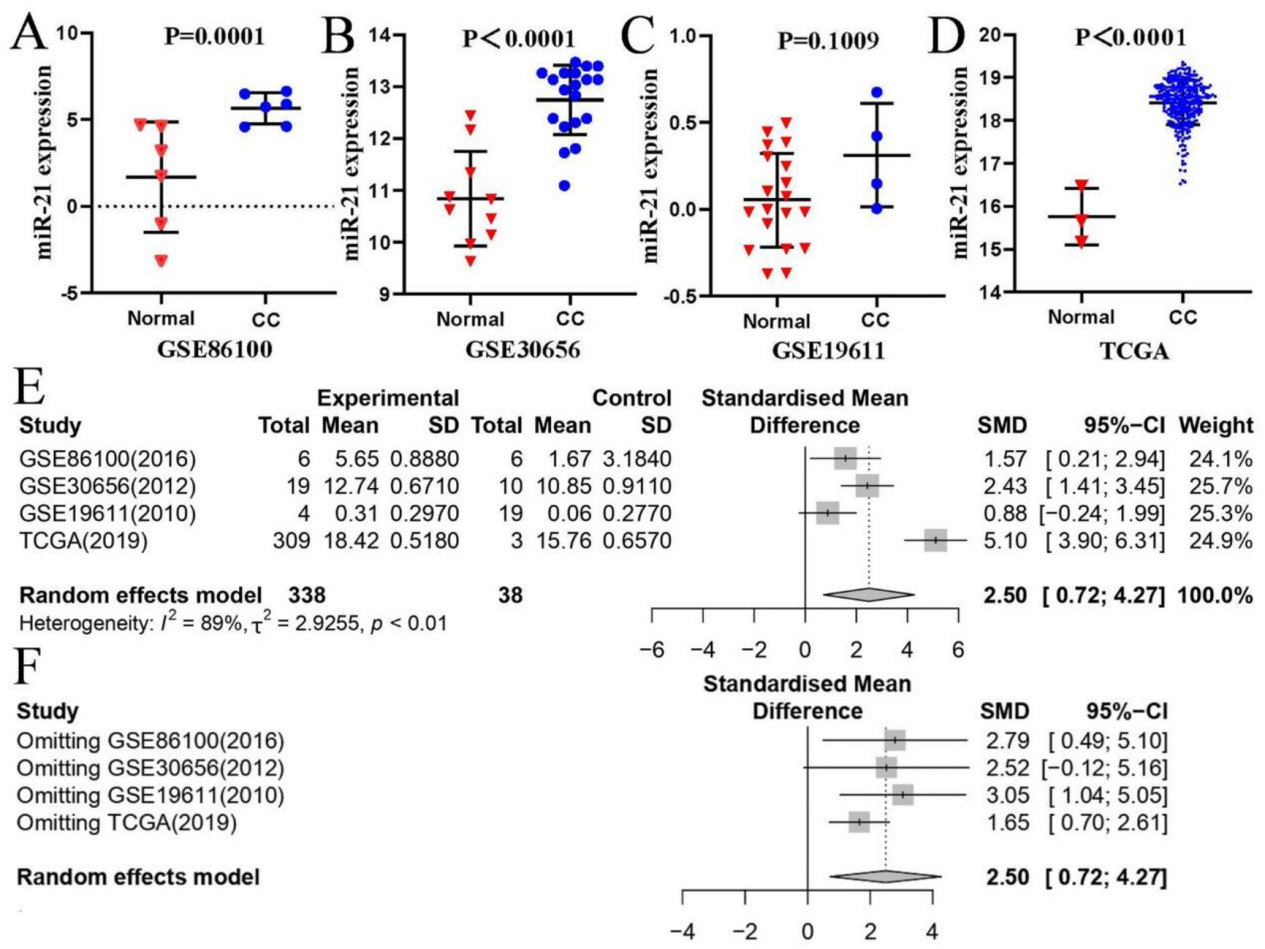
**(A-D)** Scatterplot of the miR-21 expression levels in the included cervical cancer studies. **(A)** GSE86100; **(B)** GSE30656; **(C)** GSE19611; **(D)** TCGA. **(E)** A forest plot of miR-21 expression between CC and adjacent nontumor tissues. **(F)** Sensitivity analysis of the selected four studies. Note: Normal, adjacent nontumor tissues; CESC, cervical squamous cell carcinoma and endocervical adenocarcinoma; TCGA, The Cancer Genome Atlas.

The workflow of the study is illustrated in Fig 1A, while Fig 1B displays the flow chart of the search and selection process. Eventually, 3 microarrays from GEO database (GSE86100 [22], GSE30656 [23], GSE19611 [24]) and TCGA data, including 376 cases in total, were included in this study. The specific information is displayed in Table 1. In addition, the expression data of miR-21 from the tumor tissues and adjacent noncancerous tissues (serving as control groups) are shown in Fig 2A–2D. The CC groups had a significantly higher miR-21 expression than the control groups in GSE86100, GSE30656, and TCGA (*P =* 0.0141, *P <* 0.0001, *P <* 0.001), while no notable distinction was detected in GSE19611 (*P =* 1.009).

**Table 1:** Features of the enrolled datasets.

Accession	GPL	Year	CESC			Normal			Source
			N	M	SD	N	M	SD	
GSE86100	GPL19730	2016	6	5.652	0.888	6	1.667	3.184	Tissue
GSE30656	GPL6955	2012	19	12.745	0.671	10	10.845	0.911	Tissue
GSE19611	GPL7534	2010	4	0.312	0.297	19	0.057	0.277	Tissue
TCGA	——	2019	309	18.417	0.518	3	15.761	0.659	Tissue

**Note**: TCGA, The Cancer Genome Atlas; GPL, GEO platform; CESC, cervical squamous cell carcinoma and endocervical adenocarcinoma; N, number of cases; M, Mean; SD, Standard deviation.

### 3.2 The meta-analysis of the data and the diagnostic value of miR-21

According to the results of our meta-analysis (Fig 2E), the random-effects model was applied since the degree of heterogeneity were high (*I*^*2*^ = 89%, *P* < 0.01). The combined standardized mean difference (SMD) greater than zero were considered as further supporting evidence of the higher expression of miR-21. The sensitivity analysis as showed in Fig 2F, after omitting GSE30656, the combined SMD became moot (95% CI = -0.12–5.16), which proved that our results were not stable enough.

**Fig 3:**
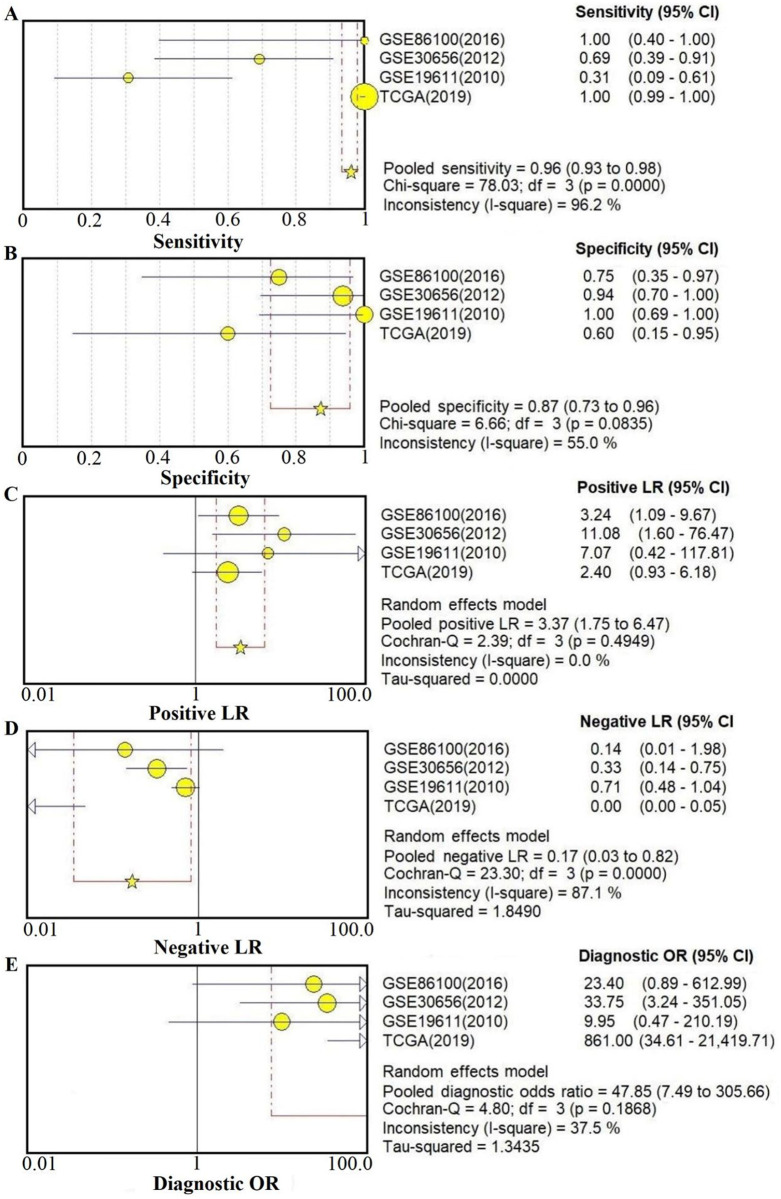
Forest plots described the diagnosis value of miR-21 of cervical cancer. **(A)** Sensitivity; **(B)** Specificity; **(C)** Positive likelihood ratio; **(D)** Negative likelihood ratio; **(E)** Diagnostic odds ratio.

**Fig 4:**
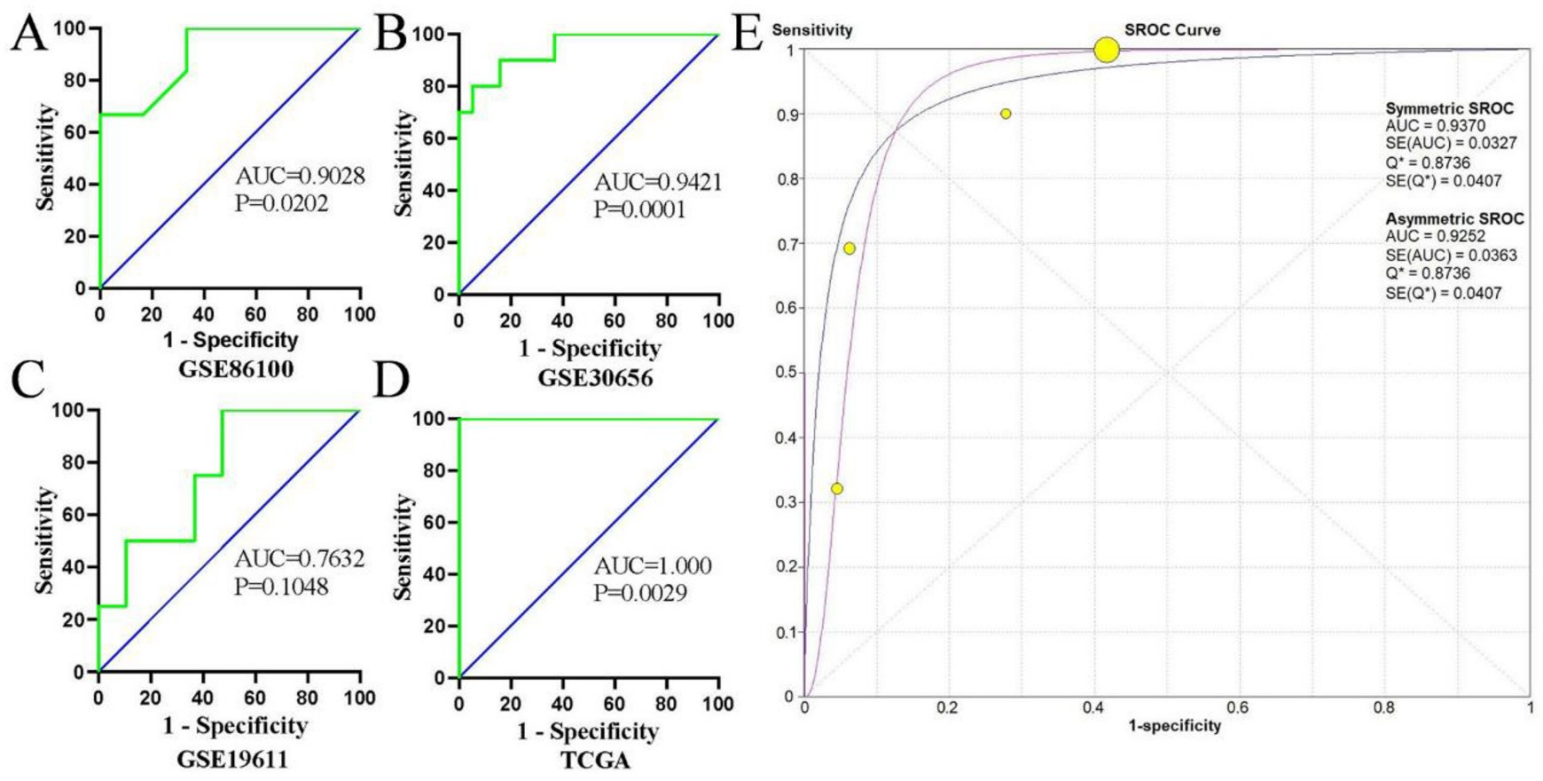
**(A-D)** Receiver operating characteristic (ROC) curves based on 4 studies. **(A)** GSE86100; **(B)** GSE30656; **(C)** GSE19611; **(D)** TCGA. **(E)** Summary receiver operating characteristic (sROC) curve for the diagnostic value of miR-21 in cervical cancer.

Next, the results of diagnostic meta-analysis included pooled specificity, sensitivity, PLR, NLR and DOR were described in Fig 3A–3E, respectively. It can be seen from Fig 4A–4E, the area under the curve (AUC) of either ROC or sROC were very high, which implied the performance of our model is good.

Furthermore, the relationship between miR-21 and clinicopathological features were also explored in TCGA database based on 309 CC samples and 3 adjacent nontumor tissues. As illustrated in Table 2, miR-21 expression in CC was much higher than that in adjacent noncancerous tissues. In addition, in patients with distant metastasis (M1), it was significantly lower than that in patients without distant metastasis (M0) (*P* < 0.05). Nevertheless, there is no statistical significance between miR-21 and other clinical pathological features.

**Table 2:** Association between miR-21 expression and clinical features from the TCGA dataset.

Variables	Terms	N	Mean ± SD	*p value*
Tissue	Adjacent noncancerous tissue	3	15.761±0.657	< 0.0001*
	CESC	309	18.417±0.518	
Pathological diagnosis	SCC	257	18.421±0.529	0.6443
	ASCA	55	18.251±0.765	
Age(years)	< 60	245	18.331±0.563	0.3353
	≥ 60	67	18.408±0.584	
Clinical grade	I-II	236	18.391±0.571	0.9344
	III-IV	69	18.397±0.633	
T stage	T1-T2	217	18.379±0.617	0.1239
	T3-T4	32	18.559±0.617	
N stage	N0	138	18.352±0.633	0.9913
	N1-N3	62	18.351±0.574	
M stage	M0	116	18.408±0.528	0.0022*
	M1	11	17.851±0.892	
Race	White	214	18.371±0.566	0.6409
	Nonwhite	62	18.331±0.672	

Note: CESC, cervical squamous cell carcinoma and endocervical adenocarcinoma; SCC, squamous cell carcinoma; ASCA, adenocarcinoma; SD, standard deviation; T stage, size or direct extent of the primary tumor; N stage, degree of spread to regional lymph nodes; M stage, presence of distant metastasis.

*p < 0.05 was considered statistically significant.

### 3.3 Identifying the promising miR-21 target genes in CC using bioinformatics

**Fig 5:**
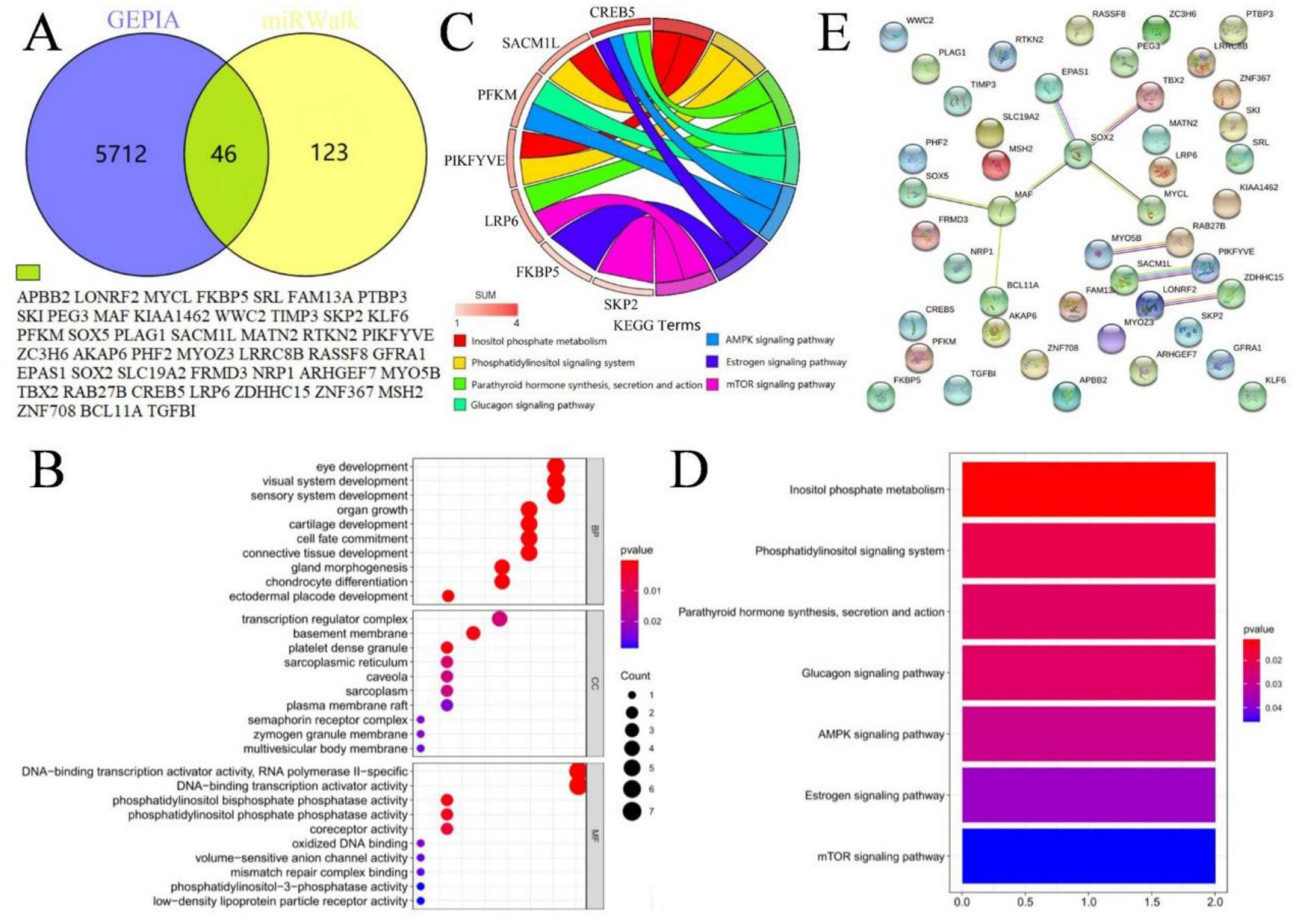
**(A)** The intersection of GEPIA2 and miRWalk2.0 to obtain predictive target genes. **(B)** Gene ontology terms categorization and distribution of the miR-21 targeted genes. **(C)** Chord plot of the KEGG pathways. **(D)** Bar graph of the KEGG pathways. **(E)** The PPI networks of the target genes of miR-21.

A total of 169 genes were obtained by more than six algorithms from miRWALK2.0, while 5758 overexpressed genes were collected from GEPIA2. After intersection, 46 predicted genes were screened (Fig 5A and S1 Table).

To further uncover the function of miR-21 in CC, KEGG and GO annotations were performed. Details were provided in Fig 5B–5D, S2 and S3 Tables. Of note, the KEGG enrichment analysis revealed that miR-21 can make a critical difference in CC through multiple pathways, including the phosphatidylinositol signaling system and mammalian target of rapamycin (mTOR) signaling pathway (Fig 5C and 5D). Fig 5E displayed the PPI network diagrams. What’s more, the top 4 hub genes (MAF, EPAS1, PIKFYVE, and SACM1 L) were calculated via Cytoscape.

### 3.4 The expression and prognostic condition of miR-21 targeted genes

**Fig 6:**
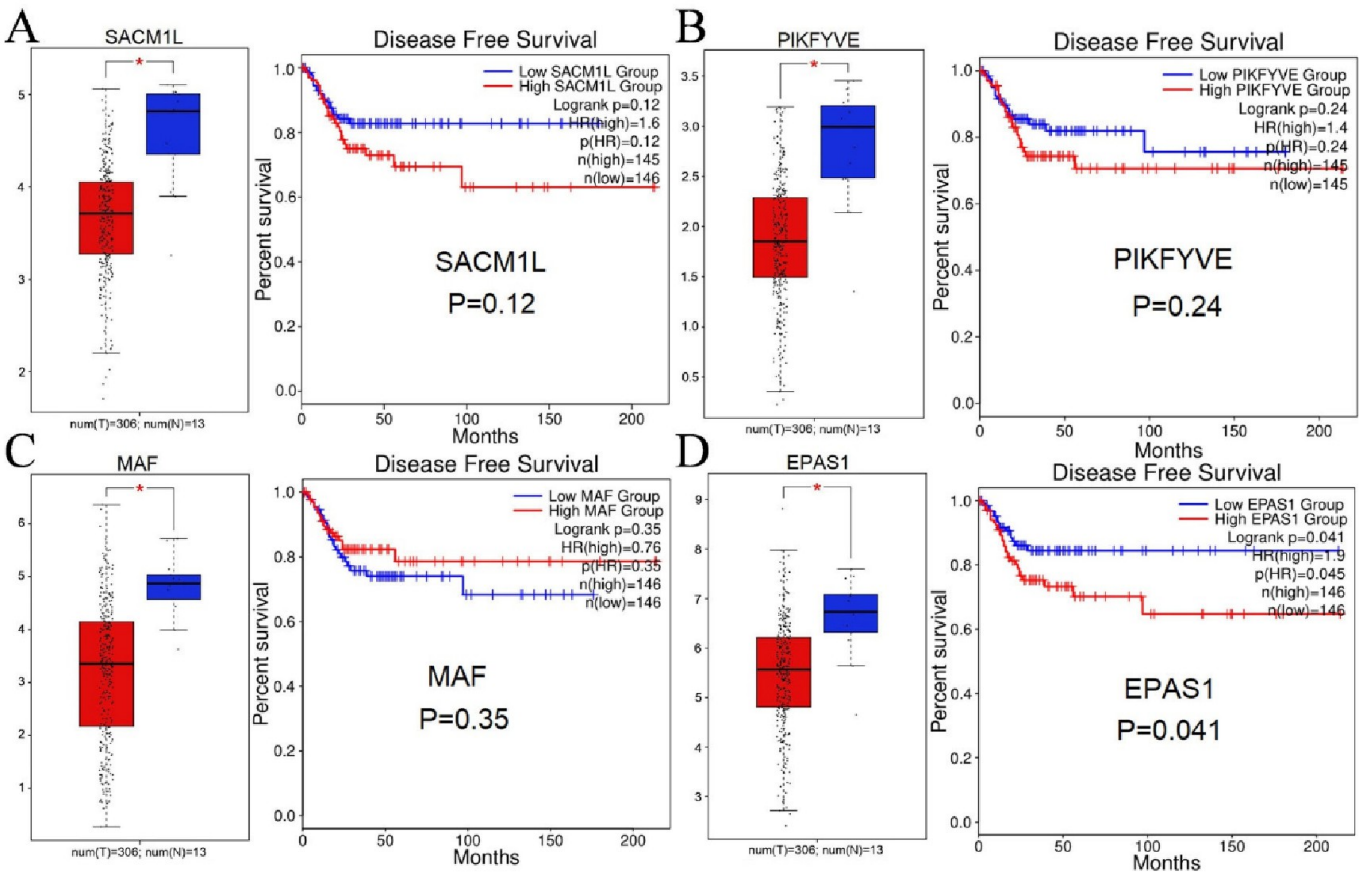
Expression level and disease-free survival curves of hub genes in CC samples and normal tissues. **(A)** SACM1 L; **(B)** PIKFYVE; **(C)** MAF; **(D)** EPAS1. Notes: Expression of the hub genes was detected in 306 cervical cancer tissues (T) and 13 adjacent nontumor tissues (N) based on the GEPIA database, with the cutoff criteria of |log_2_FC| ≥ 1.0 and adj. *p* < 0.05. SACM1 L, suppressor of actin mutations 1-like; PIKFYVE, phosphoinositide kinase, FYVE-type zinc finger containing; MAF, macrophage activating factor; EPAS1, endothelial PAS domain protein 1.

**Fig 7:**
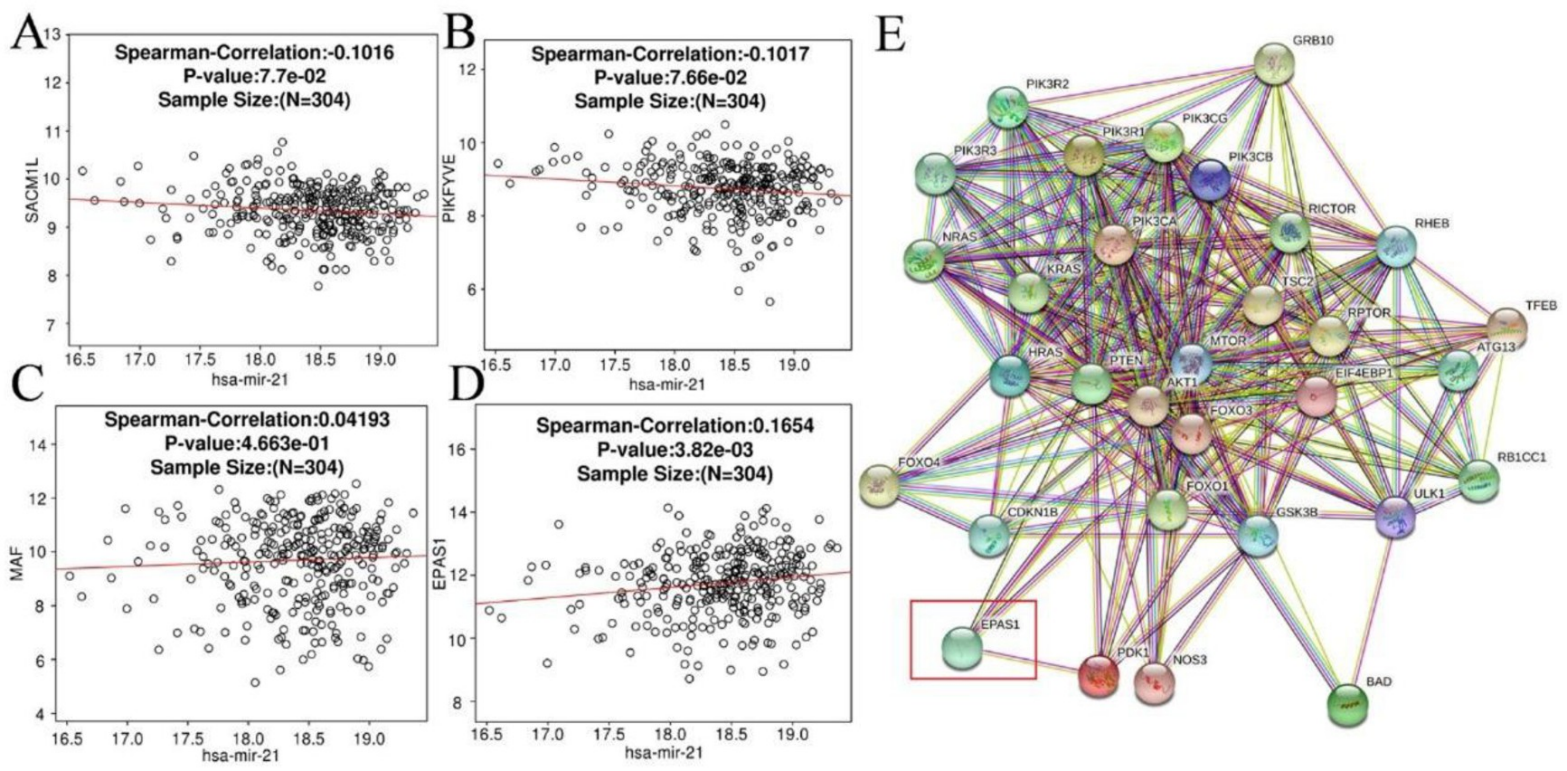
**(A-D)** Spearman’s analysis was used to show the correlations between the miR-21 expression levels and the validated target genes. **(A)** SACM1 L; **(B)** PIKFYVE; **(C)** MAF; **(D)** EPAS1. **(E)** The PPI network between EPAS1 and genes within the PI3K/AKT/mTOR signaling pathway. Notes: SACM1 L, suppressor of actin mutations 1-like; PIKFYVE, phosphoinositide kinase, FYVE-type zinc finger containing; MAF, macrophage activating factor; EPAS1, endothelial PAS domain protein 1.

Subsequently, the correlations between miR-21 and those identified hub genes were verified via LinkedOmics (http://www.linkedomics.org/).The expression of the four hub genes obtained in the previous step and their DFS curves are shown in Fig 6A–6D. From the figure, we found all four genes were significantly downregulated in the CC group compared to the control group. Notably, only EPAS1 was prognosis-related (*P* = 0.041), while the remaining 3 hub genes were not associated with an improved DFS (MAF: *P* = 0.35, PIKFYVE: *P* = 0.24, SACM1 L: *P* = 0.12). The correlation between the identified target genes and miR-21 is shown in Fig 7A–7D. Similarly, there were only EPAS1 hold a weak positive correlation (r = 0.1657) with miR-21. Given this, EPAS1 were regarded as the final target of miR-21 and used in following analyses.

According to previous GO enrichment analysis, those candidate targets are enriched in DNA-binding transcription activator activity and phosphatidylinositol phosphate phosphatase activity, both of which are associated with oncology. The KEGG analysis results revealed that mi-21 may involve in the phosphatidylinositol and the mTOR signaling pathways. One of the most prestigious phosphatidylinositol pathways is the phosphatidyl-inositol 3-kinase/serine-threonine kinase (PI3K/AKT) signaling pathway, which is implicated in the development and progression of cancers. Therefore, PI3K/AKT/mTOR was considered as a candidate signaling pathway.

In the process of validation, Table 3 illustrated that there were miR-21 binding sites both on the promotor and coding region of EPAS1 were predicted by miRactDB. Moreover, a total of 31 genes, including EPAS1 and 30 genes within the PI3K/AKT/mTOR signaling pathway downloaded from the PathCards module of GeneCards database, were applied to constructed PPI network. In Fig 7E, it’s clearly apparent that EPAS1, the final target of miR-21, were strongly interlinked with the PI3K/AKT/mTOR signaling pathway. Along this process, miR-21 and the cancer-involved pathways were contacted via EPAS1.

**Table 3:** Hsa-miR-21 binding sites on EPAS1 (promotor, coding region) predicted by miRactDB.

Gene	miRNA	Start	End	seed seq. with additional 2bp	miRNA seed seq.
miRNA binding site in promoter region of gene of plus strand (TSS +/- 2kb regions)					
EPAS1	miR-21	1090	1096	UGGGUGUUAUU	AACACCA
miRNA binding site in coding region (CDS)					
EPAS1	miR-21	1055	1061	CGUGGUGUUCU	AACACCA

## Discussion

A growing body of evidence suggested that miR-21 plays a fundamental role in migration, invasion, metastasis, and proliferation of breast cancer, head and neck squamous cell carcinoma, gastric cancer, colorectal cancer, etc. [25–29]. In this study, the tissues type (adjacent noncancerous tissue or CC samples) and the occurrence of distant tumor metastasis (M0 or M1) were associated with miR-21. This also indicated that miR-21 may be involved in the occurrence and development of CC.

Studies have examined that miR-21 may be a suitable diagnostic biomarker for colorectal cancer, with moderate sensitivity and specificity [30], while hold good diagnostic value in breast cancer and colorectal cancer [31, 32]. However, the potential diagnostic value of miR-21 in CC and its correlation with clinical features had seldom been studied. The present study focused on investigating the role and latent mechanism of miR-21 in CC by co-applying meta-analysis and bioinformatic approaches.

Here, increased expression of miR-21 was found in GSE86100, GSE30656 and TCGA, but in GSE19611, no significant differences were observed. A possible reason for this may be the limited number of tumor samples in GSE19611. Furthermore, a meaningless combined SMD emerged when sensitivity analysis was conducted (Fig 2F). We can see from Fig 2E, although the sample size of GSE30656 was not the largest one, its weighting ratio was the highest, reaching 25.7%. Given this, once GSE30656 was removed, the analysis results were severely fluctuated. In the future, higher quality and larger sample size studies need to be performed to obtain more instructive conclusions.

From Fig 2E, forest plot of the meta-analysis, the combined SMD was 2.50 (95% CI: 0.72–4.27, random-effects model). Additionally, the AUC value of either ROC or sROC were more than 0.7 (Fig 4A–4E). In a word, our meta-analysis demonstrated that miR-21 may serves as a highly sensitive and specific biomarker in CC. Subsequently, we performed a series of bioinformatic assays.

On the one hand, PI3K/AKT/mTOR was thought as a candidate signaling pathway after enrichment analyses [33–35]. What’s more, the effect of miR-21 on PI3K/AKT/mTOR pathway has also been demonstrated in other kinds of diseases. Based on the above information, it can be reasonably speculated that miR-21 may affect the invasion and migration of CC cells by regulating the PI3K/AKT/mTOR pathway.

On the other hand, EPAS1 were screened to be the final target of miR-21. EPAS1 is the only one that has statistically significant correlation with DFS and holds the largest Spearman correlation coefficients with miR-21 among the four targeted genes. Endothelial PAS domain protein 1 (EPAS1), which is also known as hypoxia-inducible factor-2α (HIF-2α), belongs to the family of hypoxia-inducible factors (HIFs) [36]. Hypoxia is a common feature of many solid tumors, including CC. The mTOR pathway, which is essential for cell proliferation, is repressed under hypoxia. As early as 2012, report showed that activation of the HIF-2α pathway increases the activity of mammalian target of rapamycin complex 1 (mTORC1) [37]. Moreover, a recent paper reported that HIF-2α could promote the apoptosis of breast cancer cells via PI3K/AKT/mTOR signaling pathway [38]. Although the function of EPAS1 in the tumorigenesis of CC is yet clear, we have demonstrated that EPAS1 were tightly linked with the PI3K/AKT/mTOR pathway. Thus, we speculated that EPAS1 plays an important role in CC through the PI3K/AKT/mTOR pathway.

It is undeniable that this study has several limitations. First, the composition of the samples differed somewhat due to the differences between data sources. For example, in the TCGA database, there were 309 cases of CC tissues and 3 cases of adjacent nontumor samples (Table 2 and Fig 2D), while the corresponding figures in the GEPIA2 database were 306 and 13, respectively (Fig 6). There is a risk that a mistake will occur, but not to the extent to which it could influence the reliability of the data, as the large data volumes from TCGA database were included. Next, because only four independent datasets were incorporated, we did not assess the risk of publication bias. Finally, due to the HPV infection status in TCGA are unavailable, we failed to identify the relationship of HPV-related CC and miR-21.

Taken together, the results of this study confirm that miR-21 is upregulated and plays a vital role in the occurrence and development of CC. More importantly, miR-21 has the potential to be a novel, highly sensitive, highly specific, noninvasive biomarker for the diagnosis of CC, but additional large-scale studies are required to verify its diagnostic value. The results of bioinformatic researches present a new method for exploring the pathogenesis of CC and will guide our experimental thinking.

## Supporting information

S1 Table Promising 41 target genes of miR-21 in CC obtained from the intersection of GEPIA2 and miRWalk2.0. (DOCX)

S2 Table The GO enrichment analysis of predicted target genes. (DOCX)

S3 Table The KEGG enrichment analysis of predicted target genes. (DOCX)
