# Supplementary material for: The clinical value of miRNA-21 in cervical cancer: A comprehensive investigation based on microarray datasets
Source: PLoS One. 2022 Apr 29;17(4):e0267108. doi: 10.1371/journal.pone.0267108 (PMC9053781; doi:10.1371/journal.pone.0267108)
Supplement: S1 Table — (DOCX) [file pone.0267108.s001.docx]

**Table S1**. Promising 41 target genes of miR-21 in CC obtained from the intersection of GEPIA2 and miRWalk2.0.

| **Sources of genes** | **Genes** | | | | | |
| --- | --- | --- | --- | --- | --- | --- |
| GEPIA and miRWalk | APBB2 | LONRF2 | MYCL | FKBP5 | SRL | FAM13A |
|  | PTBP3 | SKI | PEG3 | MAF | KIAA1462 | WWC2 |
|  | TIMP3 | SKP2 | KLF6 | PFKM | SOX5 | PLAG1 |
|  | SACM1 L | MATN2 | RTKN2 | PIKFYVE | ZC3H6 | AKAP6 |
|  | PHF2 | MYOZ3 | LRRC8B | RASSF8 | GFRA1 | EPAS1 |
|  | SOX2 | SLC19A2 | FRMD3 | NRP1 | ARHGEF7 | MYO5B |
|  | TBX2 | RAB27B | CREB5 | LRP6 | ZDHHC15 | ZNF367 |
|  | MSH2 | ZNF708 | BCL11A | TGFBI |  |  |

Note: TCGA, The Cancer Genome Atlas.
