# Supplementary material for: The clinical value of miRNA-21 in cervical cancer: A comprehensive investigation based on microarray datasets
Source: PLoS One. 2022 Apr 29;17(4):e0267108. doi: 10.1371/journal.pone.0267108 (PMC9053781; doi:10.1371/journal.pone.0267108)
Supplement: S2 Table — (DOCX) [file pone.0267108.s002.docx]

**Table S2.** The GO enrichment analysis of predicted target genes.

| GO ID | GO term | Count (%) | | Gene symbol | *P* value |
| --- | --- | --- | --- | --- | --- |
| Biological process | | | | | |
| GO:0035265 | organ growth | | 5 | WWC2/PLAG1/MATN2/AKAP6/TBX2 | 9.08E-05 |
| GO:0051216 | cartilage development | | 5 | MAF/SOX5/MATN2/LRP6/TGFBI | 0.000102 |
| GO:0001654 | eye development | | 6 | SKI/MAF/SOX2/NRP1/TBX2/LRP6 | 0.000148 |
| GO:0022612 | gland morphogenesis | | 4 | PLAG1/NRP1/TBX2/LRP6 | 0.00015 |
| Cellular component | | | | | |
| GO:0042827 | platelet dense granule | | 2 | TIMP3/RAB27B | 0.00095 |
| GO:0005604 | basement membrane | | 3 | TIMP3/MATN2/TGFBI | 0.001163 |
| GO:0016529 | sarcoplasmic reticulum | | 2 | SRL/AKAP6 | 0.010496 |
| GO:0005667 | transcription regulator complex | | 4 | SKI/EPAS1/SOX2/TBX2 | 0.012501 |
| GO:0005901 | caveola | | 2 | AKAP6/LRP6 | 0.013182 |
| Molecular function | | | | | |
| GO:0001228 | DNA-binding transcription activator activity, RNA polymerase II-specific | | 7 | MYCL/MAF/KLF6/PLAG1/EPAS1/SOX2/TBX2 | 8.21E-05 |
| GO:0001216 | DNA-binding transcription activator activity | | 7 | MYCL/MAF/KLF6/PLAG1/EPAS1/SOX2/TBX2 | 8.32E-05 |
| GO:0034593 | phosphatidylinositol bisphosphate phosphatase activity | | 2 | SACM1 L/PIKFYVE | 0.001413 |
| GO:0052866 | phosphatidylinositol phosphate phosphatase activity | | 2 | SACM1 L/PIKFYVE | 0.001948 |

**Notes**: Only the top 4 are presented in the table**.**
