# Supplementary material for: The clinical value of miRNA-21 in cervical cancer: A comprehensive investigation based on microarray datasets
Source: PLoS One. 2022 Apr 29;17(4):e0267108. doi: 10.1371/journal.pone.0267108 (PMC9053781; doi:10.1371/journal.pone.0267108)
Supplement: S3 Table — (DOCX) [file pone.0267108.s003.docx]

**Table S3**. The KEGG enrichment analysis of predicted target genes.

| KEGG ID | KEGG term | Count (%) | Gene symbol | *P* |
| --- | --- | --- | --- | --- |
| hsa00562 | Inositol phosphate metabolism | 2 | SACM1 L/PIKFYVE | 0.011237 |
| hsa04070 | Phosphatidylinositol signaling system | 2 | SACM1 L/PIKFYVE | 0.019291 |
| hsa04928 | Parathyroid hormone synthesis, secretion, and action | 2 | CREB5/LRP6 | 0.022786 |
| hsa04922 | Glucagon signaling pathway | 2 | PFKM/CREB5 | 0.02319 |
| hsa04152 | AMPK signaling pathway | 2 | PFKM/CREB5 | 0.028703 |
| hsa04915 | Estrogen signaling pathway | 2 | FKBP5/CREB5 | 0.037117 |
| hsa04150 | mTOR signaling pathway | 2 | SKP2/LRP6 | 0.045832 |
